# Does the video head impulse test replace caloric testing?^☆^

**DOI:** 10.1016/j.bjorl.2020.01.001

**Published:** 2020-01-11

**Authors:** Alessandra Ramos Venosa, Mauricio Malavasi Ganança, Raquel Mezzalira, Roseli Saraiva Moreira Bittar, Fernando Freitas Ganança

**Affiliations:** a Universidade de Brasília (UnB), Faculdade de Medicina (FM), Brasília, DF, Brazil; b Universidade Federal de São Paulo (Unifesp), Escola Paulista de Medicina (EPM), São Paulo, SP, Brazil; c Universidade Estadual de Campinas (Unicamp), Disciplina de Otorrinolaringologia e Cirurgia de Cabeça e Pescoço, Campinas, SP, Brazil; d Universidade de São Paulo (USP), Faculdade de Medicina (FM), Hospital das Clínicas (HC), Setor de Otoneurologia, São Paulo, SP, Brazil; e Universidade Federal de São Paulo (Unifesp), Escola Paulista de Medicina (EPM), Disciplina de Otologia e Otoneurologia, São Paulo, SP, Brazil

We have received recurring questions from colleagues and students about the substitution of the caloric test (CT) by the video impulse test (vHIT). Two reasons are often used to justify the change: 1) the CT dates from the early twentieth century and 2) the test is uncomfortable; the vHIT, in turn, is a recent test and causes minimal discomfort. As scientists, we must state our opinions always based on physiological foundations.

The CT was described in the early twentieth century by Robert Bárány, who received the Nobel Prize for his achievement, considering its clinical importance for the diagnosis of vestibular diseases.^1^ It remains the only functional test capable of isolating the stimulated side. The head impulse test (HIT), described at the end of the century, is a remarkable demonstration of how to easily and reliably observe the vestibulo-ocular reflex (VOR) performance.^2^ The documentation by computer programs came after the clinical description in both cases. The fact that one test was described before and the other after does not interfere with its clinical indication and applicability.

Regarding the discomfort caused by the test, the argument does not resist a more detailed assessment. As an example, one can report cases of migraine patients in whom both CT and vHIT cause great discomfort, sometimes triggering the crisis on subsequent days. Some do not even tolerate the vHIT mask, let alone the cephalic impulses. However, most patients tolerate both tests well. What drives the test indication is its benefit in attaining the final diagnosis^3^ and not whether it is uncomfortable or not. Or does anyone doubt that a mammogram is a fundamental diagnostic exam, although highly painful and uncomfortable?

There have been discussions regarding the stimulation mechanism caused by CT in the labyrinth cells. Since its description, there have been hypotheses about how the temperature acts on the ampullary crest and inferences about the stimulated cell type. There are still doubts as to whether the result is caused by functional, structural damage or even hair cell integrity. That is a fact, and much remains to be studied, but the question that really matters is what CT adds to the final diagnosis.

With the increase in practical knowledge, we now know that in some cases, such as in Meniere's Disease, the alteration in the CT together with a normal vHIT is highly suggestive of the diagnosis.^4^ This is what matters: how much these data can help you establish the best way to manage your patient.

Although these are two forms of VOR documentation, the stimuli used are different. In CT, the stimulus is the variation in temperature and in the vHIT, the stimulus is mechanical (cephalic impulse). Both methods have limitations. In CT, the stimulus is unilateral and of low frequency and in the vHIT, the stimulation is bilateral and occurs in response to higher velocities. The high-velocity impulses in vHIT are necessary to isolate the vestibular reflex, while at low frequencies it is influenced by visual information. The examination allows documenting vertical movements, although these records are more subject to artifacts.

We see the tests as distinct evaluations to obtain response from ampullary crests – functional units of angular movements.^5^ The tests complement each other and may be ordered together or separately, depending on their usefulness for attaining the diagnosis.

If the question is “Does the vHIT replace the CT”, the answer is no. It is up to the physician to choose which test should be requested for their patient, introduced in their diagnostic routine and whether they will be submitted to one or both, which will be carried out before or after. There is no right or wrong: the way the physician is more comfortable when performing his diagnostic evaluation should guide the choice of diagnostic testing.

## Conflicts of interest

The authors declare no conflicts of interest.
